# Synchronous Operable Pancreatic and Breast Cancer Without Genetic Mutation: A Literature Review and Discussion

**DOI:** 10.3389/fsurg.2022.858349

**Published:** 2022-06-24

**Authors:** Adam Ofri, Danika Zuidersma, Connie I. Diakos, Amanda Stevanovic, Matthew Wong, Samriti Sood, Jaswinder S. Samra, Anthony J. Gill, Anubhav Mittal

**Affiliations:** ^1^ Department of Surgery, Sir Charles Gairdner Hospital, Nedlands, Australia; ^2^ St Vincent’s Clinical School, University of New South Wales, Sydney, Australia; ^3^ Kolling Institute of Medical Research, University of Sydney, Sydney, Australia; ^4^ Department of Medical Oncology, Royal North Shore Hospital, St Leonards, Australia; ^5^ Department of Medical Oncology, Nepean Cancer Care Centre, Nepean, Australia; ^6^ Central Coast Cancer Centre, Gosford Hospital, Gosford, Australia; ^7^ Department of Breast Surgery, Royal North Shore Hospital, St Leonards, Australia; ^8^ Upper GI Surgical Unit, Royal North Shore Hospital and North Shore Private Hospital, St Leonards,Australia; ^9^ Australian Pancreatic Centre, St Leonards, Sydney, Australia; ^10^ Northern Clinical School, Faculty of Medicine and Health, University of Sydney, Sydney, Australia; ^11^ School of Medicine, Macquarie University, Sydney, Australia; ^12^ NSW Health Pathology, Department of Anatomical Pathology, Royal North Shore Hospital, St Leonards, Australia; ^13^ School of Medicine, University of Notre Dame, Sydney, Australia

**Keywords:** pancreatic cancer, breast cancer, synchronous, non-germline, neoadjuvant treatment

## Abstract

**Background:**

Synchronous cancers are rarely detected when working-up a patient for a primary cancer. Neoadjuvant management of synchronous breast and pancreatic cancers, without a germline mutation, has yet to be discussed. Two patients were diagnosed with synchronous breast and pancreatic cancers at our institution over the last decade. A literature review was performed to evaluate the current evidence stance.

**Results:**

The first patient was 61-years old and diagnosed with a HER2+ breast cancer. The second patient was 77-years old and diagnosed with a Luminal B breast cancer. The inability to provide concurrent breast and pancreatic neoadjuvant therapy for the HER2+ patient, resulted in upfront surgery. The second patient was able to have both cancers treated simultaneously - neoadjuvant chemotherapy to the pancreas, and neoadjuvant endocrine therapy to the breast.

**Discuss:**

There is no single neoadjuvant regimen that treats both pancreatic and breast cancer. The differences in breast cancer sub-types impacted our neoadjuvant options. Our recent experience led us to the hypothesis that breast cancer care dictates treatment, while pancreatic cancer determines survival. There is a significant paucity in the literature regarding synchronous breast and pancreatic cancer.

## Introduction

The management of operable pancreatic cancer patients is complex and multifaceted. The typical pancreatic cancer presentation is late, with less than 20% up-front surgical candidates. The term “borderline” resectable has been used with varying definitions. The underpinning notion being that surgery is feasible, however the likelihood of incomplete resection is high (1–3). For these cases, downstaging with neoadjuvant and repeat staging is currently recommended (4). Dual malignancy can significantly complicate the management of patients.

The presence of synchronous pancreatic and breast cancers is rare, having been described in a handful of case reports, resulting in management difficulty (5, 6). Over the last decade, the genetics associated with pancreatic cancer has been intricately assessed (7) Some well-known cancer gene germline mutations have been identified in patients with pancreatic cancer, including BRCA1/2, PALB2, STK11, PRSS1, SPINK1 and ATM. Pathogenic mutations are identified in approximately 15% of pancreatic adenocarcinoma patients; with germline BRCA mutations accounting for more than 50% of these mutations (8). Somatic mutations in the homologous recombination pathway mimicking germline BRCA1/2 loss are termed “BRCAness” or “BRCA-like” genes (9, 10). However, it is uncertain whether germline mutation treatments are as effective (11, 12).

BRCA1 and BRCA2 are associated with the DNA repair pathway and together, are the most common genetic cause for breast cancer, accounting for approximately 5% of all cases. Patients with BRCA1/2 mutations have a 72% and 69% lifetime risk of developing breast cancer respectively, with increased ovarian cancer risks. BRCA1 also increases the relative risk (RR) for pancreatic cancer by 2 to 4-fold, where BRCA2 increases RR by 3 to 8-fold (13).

The 5-year survival rate for breast cancer in Australia is approximately 91% (14). Nodal status greatly impacts 5-year survival rates, 96% for node-negative, 80% for node positive. Comparatively, pancreatic cancer is an aggressive malignancy with a reported Australian 5-year survival rate of 10.7% (15). Given the significantly different survival rates, one would expect that pancreatic cancer management would drive patient care.

However, our recent experience led us to the hypothesis that breast cancer care may dictate treatment, while pancreatic cancer determines survival. Here we present our two cases and review the current extent of literature for synchronous breast and pancreatic cancers.

## Methods

From January 2020 until January 2021, two patients were diagnosed with synchronous pancreatic and breast cancers in the Upper Gastrointestinal Surgery Department of Royal North Shore Hospital, NSW, Australia, a tertiary referral centre for pancreatic cancer care. Ethical approval was obtained from the Northern Sydney Local Health District Human Research and Ethics Committee. Of the two patients, one underwent upfront surgery and the other received neoadjuvant chemotherapy. Given the rarity of synchronous pancreatic and breast cancer, a comprehensive literature review was performed. A literature search was performed using the PubMed, EMBASE, Medline, Web of Science, and Cochrane Library databases for studies published between 1 January 1990 and 31 August 2021. The following MeSH terms and their combinations were searched: (breast +/− cancer/neoplasm/tumor/tumour) and (pancreas/pancreatic +/− cancer/neoplasm/tumor/tumour) and (synchronous). Peer-reviewed papers with English abstracts were all reviewed. Two authors (AO & DK) independently reviewed the titles and abstracts to screen and extract relevant articles.

## Results

### Case 1 – Surgery First

A 61-year-old woman presented with ongoing epigastric pain post cholecystectomy. An MRI scan identified an intraductal papillary mucinous neoplasm (IPMN). Due to significant patient anxiety and initial refusal for an endoscopic ultrasound (EUS), an FDG-PET was performed, identifying FDG uptake in a lesion in the pancreatic head, as well as a in a lesion in the left breast and two left axillary lymph nodes. An MRI scan of the breast noted a 37 mm irregular, spiculated lesion in the upper outer quadrant of the left breast. CNB of the breast lesion confirmed Grade 3 IDC, hormone receptors negative, amplified for HER-2, with a Ki67 of 40%. Axillary lymph node FNAB was positive for breast carcinoma. EUS of the pancreas identified a 1.5 cm mass in the neck of the pancreas and a lesion in the tail of the pancreas, with FNA confirming PDAC in both lesions. Full mutation search for BRCA1/2 and PALB2 were negative.

This case was discussed at both the Breast and Pancreas multi-disciplinary meetings, with the consensus recommendation made for up-front surgery with adjuvant chemotherapy. In this scenario, there were two aggressive malignancies with no significant overlap of active systemic treatments that would provide benefit in both cancers; therefore, the concern was for potential progression of one or both malignancies during a period of neoadjuvant systemic treatment, that would ultimately render one or both malignancies, inoperable.

The patient underwent an uneventful total pancreatectomy and simultaneous mastectomy and axillary nodal clearance. Histopathology confirmed two completely excised cancers - a Stage IIa pancreatic ductal adenocarcinoma with 1/29 lymph nodes, and a 19 mm breast cancer Grade 3 IDC with 23/25 positive axillary nodes. She was subsequently treated with Carboplatin, Paclitaxel and trastuzumab as adjuvant systemic treatment; this regimen was selected given evidence of activity in both malignancies for the chemotherapy component, with the trastuzumab directed towards the Her2-positive early breast cancer (EBC).

Unfortunately, a PET performed 5 months post operatively indicated pancreatic recurrence in the middle of the pancreatic bed, a poor prognostic indicator. She is currently undergoing chemotherapy with the FOLFIRINOX (oxaliplatin, irinotecan and 5-FU) regimen, with RT to the pancreatic bed, as salvage intent treatment.

### Case 2 – Neoadjuvant Chemotherapy First

The second patient was a 77-year-old woman, referred from her gastroenterologist after being investigated for weight loss, anorexia, and abdominal pain over 6 months. CT imaging identified an ill-defined hypodense mass at the junction of the body and neck of the pancreas with abnormally appearing lymph nodes, as well as left axillary lymphadenopathy. Breast ultrasound identified 2 lesions in the left breast, irregularly hypoechoic and in close proximity: 19 and 8 mm in size. CNB of the breast lesions confirmed Grade 2 IDC that was hormone receptor positive, HER-2 negative on IHC, with a Ki67 of 60%. The patient’s family history was unremarkable and genetic testing was negative for any known mutations.

The multi-disciplinary team recommended neoadjuvant chemotherapy directed towards the pancreatic malignancy with neo-adjuvant endocrine therapy for the breast cancer. She was commenced on the FOLFIRINOX chemotherapy regimen and Letrozole (an aromatase inhibitor, given her post-menopausal state).

She tolerated her chemotherapy regimen well. Her immediate post-chemotherapy PET-CT indicated resolution of FDG uptake in the breast and the axillary nodes, with reduction in FDG avidity to the pancreas. During her work up for operative intervention, she developed increasing Ca 19.9 levels, with a repeat PET-CT indicated liver metastases. Subsequently she was re-started on further chemotherapy and not deemed a surgical candidate.

## Discussion

We present here a series of two women presenting with synchronous operable breast and pancreatic malignancies, to a specialist multi-disciplinary quaternary referral centre. The management of these two cases differed, predominantly due to the breast pathology. In the first case, the inability to provide appropriate dual-spectrum neoadjuvant treatment (due to HER-2 amplification) resulted in up-front surgery. In the second case, due to the hormone receptor positivity, we were able to provide concurrent pancreatic-NACT and neoadjuvant endocrine treatment (NET) to the breast. Unsurprisingly, the outcomes of both cases were determined by the progression of the pancreatic adenocarcinoma, which is typically the more aggressive of the two tumour types.

### Literature Review

After a comprehensive review of the literature, only 6 studies were found to have discussed synchronous pancreatic and breast cancer (Table 1). Two papers were in Japanese with English abstracts (16, 17). Both described synchronous breast and pancreatic cancer, with one case where the pancreatic cancer had metastasized to the liver and peritoneum. A case report by Kim et al., discussed a patient diagnosed with breast and pancreatic synchronous cancers, as well as concurrent thyroid and gastrointestinal stromal cancer (18). One letter to the editor discussed a patient with prostatic cancer, who then developed primary breast and pancreatic cancer, as well as pulmonary and bone metastasis of unknown origin (20). Unek et al. reported on a male patient who developed synchronous breast and pancreatic cancer, on a background of previously treated testicular cancer (19). The only germline case report has been by Castro et al. who discussed synchronous breast and pancreatic cancer in a BRCA2 mutation (5).

**Table 1:** Summary of 6 papers discussing synchronous breast and pancreatic cancers.

Authors, Year	Case	Breast Cancer	Pancreatic cancer	Treatment	Outcome
Nakanishi et al (16), 2021	78y female - synchronous left breast cancer and pancreatic cancer	48 mm IDC, with nodal involvement, NFI (T2N1M0)	30 mm borderline resectable pancreatic body adenocarcinoma	1. NAC: gemcitabine and nab-paclitaxel. Stable breast cancer and partial response to pancreatic cancer 2. Pancreatosplenectomy with portal vein and gastroduodenal artery resection and reconstruction + left mastectomy with axillary lymph node dissection 3. Flurouracil, epirubicin and cyclophosphamide	No recurrence noted
Takada et al (17), 2018	67y male - synchronous left breast and pancreatic cancer (with liver and peritoneal metastases)	28 mm, IDC, TNBC (T2N0M0)	40 mm unresectable pancreatic tail cancer	1. Palliation due to hepatic failure secondary to pancreatic tumour growth	Died
Castro et al (5), 2016	41y female - synchronous locally advanced left breast cancer and pancreatic cancer, BRCA2 mutation	75 mm Grade 2 IDC (ER/PR+, HER2-) with HG-DCIS, 8/23 LN (T3N2aM0)	26 mm resectable pancreatic tail adenocarcinoma	1. Left modified radical mastectomy 2. Laparoscopic, hand assisted splenectomy, distal pancreatectomy, lymphadenectomy and bilateral salpingo-oophorectomy 3. Adjuvant chemotherapy: doxorubicin-cisplatin followed by gemcitabine-NAB paclitaxel 4. Radiotherapy (post-mastectomy and upper abdominal) + aromatase inhibitor 5. For consideration of Olaparib therapy	Alive at time of publication
Kim et al (18), 2013	73y female - synchronous left breast cancer, pancreatic cancer, papillary carcinoma of the thyroid and GIST.	Multiple IDC, NFI (TxNxMx)	18 mm unresectable pancreatic body adenocarcinoma with ductal dilatation and coealic axis invasion	1. Patient refused aggressive treatment including surgery 2. Agreed for treatment of the breast cancer and GIST (hormone therapy and chemotherapy): Letrozole + imatinib 3. Supportive treatment for thyroid and pancreatic cancer	Died 8 months after diagnosis
Unek et al (19), 2008	50y male - synchronous right breast cancer and pancreatic cancer. Background of previously treated mixed germ cell testicular tumour 15 years prior	11 mm Grade 1 IDC, ER+, PR-, HER2-, LVI+ (T4bNxM0)	25 mm resectable pancreatic head adenocarcinoma	1. Surgery: Whipple procedure and right mastectomy 2. Radiotherapy + Chemotherapy - Radiotherapy: to pancreatic region and chest wall - Chemotherapy: 5-Fu + Gemcitabine. Patient refused after 6 cycles due to metastatic spread	Died 10 months after diagnosis
Morganti et al (20), 2008	69y male - 2 primary right breast cancers and primary pancreatic cancer with liver metastasis Two months post starting therapy for prostatic adenocarcinoma	25 mm + 15 mm Grade 2 IDC, 0/25 LN (T2N0M0)	30 mm unresectable pancreatic body and tail adenocarcinoma with vascular invasion with liver metastases	1. Total right mastectomy 2. Chemotherapy: carboplatin-taxotere, 15 cycles 3. Zolidronic acid, monthly, 8 cycles 4. LHRH for prostate cancer continued 5. Chemotherapy second regimen due to disease progression: gemcitabine and oxaliplatin 6. Worsening ascitic effusion with pain, dyspnoea and haematologic toxicity. Regimen changed to gemcitabine alone	Died 17 months after diagnosis

*5-Fu, Fluorouracil; ER, estrogen receptor; GIST, Gastrointestinal Stromal Tumour; HG-DCIS, high-grade ductal carcinoma in-situ; IDC, invasive ductal carcinoma; LN, lymph nodes; LVI, lymphovascular invasion; NAC, neoadjuvant chemotherapy; NFI, no further information; PR, progesterone receptor; TNBC, triple negative breast cancer*.

Of the available literature, only one case discussed NACT. This was in the regimen of gemcitabine and nab-paclitaxel. Adjuvant chemotherapy varied in the other 3 cases; doxorubicin-cisplatin followed by gemcitabine-NAB paclitaxel, 5-Fu and Gemcitabine, and carboplatin-taxotere. There was no discussion regarding the decision making behind any of the chemotherapy regimens in any of the papers.

### Neoadjuvant Chemotherapy Indications

At our institution, the default pancreatic cancer management sequence is neoadjuvant chemotherapy followed by surgery for tumours >2 cm and/or node positive. Current international guidelines are varied regarding this stance. The National Comprehensive Cancer Network (NCCN) suggest NACT usage in high-risk potentially resectable (borderline) tumours, however the American Society of Clinical Oncology (ASCO) guidelines suggest against NACT if tumours are potentially resectable (21, 22). The European Society for Medical Oncology (ESMO) guidelines do not discuss NACT in potentially resectable Pancreatic cancers (23).

There are currently multiple indications for neoadjuvant breast cancer treatment– ranging from receptor status, tumour size, nodal involvement, as well as desire to perform breast conservation therapy (24–27). NACT can result in decreasing tumour size to facilitate breast conservation therapy (BCT), potentially de-escalate axillary surgery, and maximize the potential aesthetic outcomes dependent on tumour location. Landmark studies, the National Surgical Adjuvant Breast and Bowel Project (NSABP) B-18 and B-27, did not identify any statistically significant benefit for neoadjuvant chemotherapy (NACT) compared to adjuvant CTX (28). However, they have shown that NACT patients that achieved a complete pathological response (pCR) have statistically superior disease-free survival (DFS) and overall survival (OS).

### Neoadjuvant Chemotherapy Regimens

Our preferred chemotherapy regimen in pancreatic cancer is FOLFIRINOX, or a modified FOLFIRINOX, dependent on a patient’s European Cooperative Oncology Group (ECOG) performance status. The preference for FOLFIRINOX over Gemcitabine ± capecitabine is based off adjuvant studies that have shown superior survival rates for FOLFIRINOX compared to Gemcitabine, and from neoadjuvant FOLFIRINOX studies showing higher rates of R0 resections and favourable median overall survival (29, 30).

Current breast cancer NACT typically involves a regimen containing an anthracycline and taxane (31). In patients whom anthracyclines are contraindicated, a regimen of CMF can be offered. From this backbone, additions are recommended depending on the breast cancer subtype. In HER-2 positivity, the addition of HER-2 directed therapy, trastuzumab +/- pertuzumab, have been shown to significantly improve pCR, event free survival and overall survival (32, 33). In triple negative breast cancer (TNBC) the addition of carboplatin is recommended, weighing the risk to benefit.

For hormone receptor positive patients, neoadjuvant endocrine therapy (NET) has shown similar response rates, and rates of BCT, compared to NACT in post-menopausal women, with less side effects (34). In post-menopausal women, when comparing CTX with AI monotherapy (exemestane, anastrozole) – there is comparable median time until clinical response, similar pCR rates and no difference in LRR (35). Survival data at this stage though is not available. The data available regarding its efficacy in pre-menopausal is limited but suggests worse response rates relative to CTX (36). As such, current guidelines advocate for NET in pre-menopausal women in a clinical trial setting only.

In HER-2 breast cancer patients ≥ T1c, NACT is recommended with sequential trastuzumab. In our patient the delivery of NACT with HER-2 directed therapy, typically AC-T and Trastuzumab, was inappropriate due to the lack of pancreatic treatment. The inability to treat the pancreatic and breast malignancy concurrently resulted in the necessity for upfront surgery. Trastuzumab can be delivered with FOLFIRINOX, as in HER2+ gastric cancers, however FOLFIRINOX has not shown any efficacy in breast cancer. Synchronous pancreatic and breast cancers, though rare, has had literature identifying breast cancer progression whilst on FOLFIRINOX for metastatic pancreatic cancer (37).

Directed HER-2 therapy alone is not appropriate, as shown in the KRISTINE trial (38). This randomized, multi-centre, phase 3 trial failed to prove comparative pCR rates for HER-2 targeted treatment alone (trastuzumab emtansine plus pertuzumab) to traditional NACT plus dual HER-2 targeted treatment (docetaxel, carboplatin, trastuzumab and pertuzumab).

### Neoadjuvant Treatment Adjuncts in Breast Cancer

Directed management of HR-positive, HER2-negative early breast cancer, relies on anti-estrogen therapy. In advanced HR-positive, HER2-negative breast cancers, the addition of cyclin-dependent kinases 4 and 6 (CDK4/6) inhibitors has become the new standard of care. Their usage in EBC though has had mixed results (39–42) Two recent studies have shown statistically significant positive outcomes involving the usage of Abemaciclib, in both neoadjuvant and adjuvant delivery (43, 44). The monarchE study was a phase 3 study that evaluated adjuvant Anastrozole ± Abemaciclib, in post-menopausal HR-positive, HER2-negative patients with EBC. Results demonstrated superior 2-year invasive disease-free survival with the addition of Abemaciclib (92.2% versus 88.7%, *p* = 0.01).

The neoMONARCH phase 2 study compared two-week usage of Abemaciclib alone, Anastrozole alone or Abemaciclib and Anastrozole, in post-menopausal women in the neoadjuvant setting. The primary end point, Ki67 expression change, was elevated in treatment arms including Abemaciclib (Abemaciclib alone 91%, Abemaciclib and Anastrozole 93%, Anastrozole alone 63%). A complete pathological response (pCR) was achieved in 4% of the cohort that received Abemaciclib and Anastrozole. Further neoadjuvant studies are required however the potential addition of Abemaciclib to ET and would improve our management options in synchronous cancer patients.

### Potential Neoadjuvant Chemotherapy Alternatives

The combination of Gemcitabine plus nanoparticle albumin-bound (nab)-Paclitaxel (GA) have been evaluated as first line regimens for both metastatic pancreatic adenocarcinoma and breast cancer. In both cancer sub-sets, the efficacy of the combination chemotherapy has been proven. However, they are not indicated as first line treatments in either cancer.

In pancreatic adenocarcinoma, a case series compared 485 consecutive patients receiving either FOLFIRINOX or GA as first line treatment (45). They noted FOLFIRINOX was associated with higher partial response evaluation criteria in solid tumours (RECIST) rates (19% versus 6%, *p* = 0.001). In propensity-score matched analysis, FOLFIRINOX patients had higher response rates (19% versus 6%, *p* = 0.001) and were more likely to undergo pancreatectomy (29% vs 18%, *p* = 0.02). Overall survival though, was comparable between either chemotherapy regimen.

Gemcitabine has limited use in the metastatic breast cancer setting. The current indication for its use (Gemcitabine and carboplatin) is relegated to patients who have already had first line treatment (anthracycline and taxane), or to those for whom there is a contraindication to an anthracycline and/or taxane only (46). The addition of Gemcitabine to standard anthracycline and taxane chemotherapy has been evaluated, and failed to show any improvement in 5-year DFS or OS (47).

## Conclusion

Synchronous breast and pancreatic breast cancers are rare, with literature limited to case reports only currently. Neoadjuvant therapy in both cancers, has resulted in significant improvements in the outcomes for patients in each individual cancer. However, there is no single neoadjuvant regimen that is shown to completely manage both. From our experience, we have noted that the typically less aggressive of the two cancers, the breast cancer, can direct our management due to the wider scope of treatment. In Luminal breast cancers, we can provide NET concurrently with our pancreatic chemotherapy regimen. However, in HER2+ or TNBC, the inability to provide any concurrent treatment to the breast results in our requirement of upfront surgery. Given how rapidly breast cancer management is evolving, we hope that further biologic treatments would wide our treatment options even greater in these complex synchronous cancer situations. However, given the paucity of the data, further research is required to determine whether the breast pathology, rather than pancreatic, should direct treatment.

## Data Availability

The datasets presented in this article are not readily available because The data is not anonymized given the 2 complex cases. Requests to access the datasets should be directed to Adam Ofri, adamofri@gmail.com.

## References

[1] Toesca DAS Koong AJ Poultsides GA Visser BC Haraldsdottir S Koong AC Management of borderline resectable pancreatic cancer. Int J Radiat Oncol Biol Phys. (2018) 100(5):1155–74. 10.1016/j.ijrobp.2017.12.287 29722658

[2] Chawla A Molina G Pak LM Rosenthal M Mancias JD Clancy TE Neoadjuvant therapy is associated with improved survival in borderline-resectable pancreatic cancer. Ann Surg Oncol. (2020) 27(4):1191–200. 10.1245/s10434-019-08087-z 31802297

[3] Heinrich S Besselink M Moehler M Van Laethem J-L Ducreux M Grimminger P Opinions and use of neoadjuvant therapy for resectable, borderline resectable, and locally advanced pancreatic cancer: international survey and case-vignette study. BMC Cancer. (2019) 19(1):1–9. 10.1186/s12885-019-5889-5 31288786 PMC6617881

[4] Balaban EP Mangu PB Khorana AA Shah MA Mukherjee S Crane CH Locally advanced, unresectable pancreatic cancer: American society of clinical oncology clinical practice guideline. J Clin Oncol. (2016) 34(22):2654–68. 10.1200/JCO.2016.67.5561 27247216

[5] Castro M Vierkoetter K Prager D Montgomery S Sedgwick K. Synchronous onset of breast and pancreatic cancers: results of germline and somatic genetic analysis. Case rep oncol. (2016) 9(2):387–94. 10.1159/000447348 27721756 PMC5043351

[6] Erikoğlu M Şimşek G Dereli Ç Tavlı Ş. Synchronous pancreas adenocarcinoma and breast infiltrative ductal carcinoma. Electron J Gen Med. (2014) 11(Suppl. 1):51–3. 10.15197/sabad.1.11.32

[7] Biankin AV Waddell N Kassahn KS Gingras MC Muthuswamy LB Johns AL Pancreatic cancer genomes reveal aberrations in axon guidance pathway genes. Nature. (2012) 491(7424):399–405. 10.1038/nature11547 23103869 PMC3530898

[8] Salo-Mullen EE O’Reilly EM Kelsen DP Ashraf AM Lowery MA Yu KH Identification of germline genetic mutations in patients with pancreatic cancer. Cancer. (2015) 121(24):4382–8. 10.1002/cncr.29664 26440929 PMC5193099

[9] Wong W Raufi AG Safyan RA Bates SE Manji GA. BRCA mutations in pancreas cancer: spectrum, current management, challenges and future prospects. Cancer Manage Res. (2020) 12:2731–42. 10.2147/CMAR.S211151 PMC7185320 32368150

[10] Lord CJ Ashworth A. BRCAness revisited. Nat Rev Cancer. (2016) 16(2):110–20. 10.1038/nrc.2015.21 26775620

[11] Binder KAR Mick R O'Hara M Teitelbaum U Karasic T Schneider C Abstract CT234: A Phase II, single arm study of maintenance rucaparib in patients with platinum-sensitive advanced pancreatic cancer and a pathogenic germline or somatic mutation in BRCA1, BRCA2 or PALB2. AACR (2019). 10.1200/JCO.21.00003 33970687

[12] Golan T Varadhachary GR Sela T Fogelman DR Halperin N Shroff RT Phase II study of olaparib for BRCAness phenotype in pancreatic cancer. Am Soc Clin Oncol. (2018) (Suppl. 4):297. 10.15197/sabad.1.11.32

[13] Pilarski R. The role of BRCA testing in hereditary pancreatic and prostate cancer families. Am Soc Clin Oncol Educ Book. (2019) 39:79–86. 10.1200/EDBK_238977 31099688

[14] Mothoneos J Gee J. Understanding breast cancer: a guide for people with cancer, their families and friends. Cancer Council of New South Wale: Cancer council of NSW (2020).

[15] Health, A.I.O. and Welfare. Cancer data in Australia. Canberra: AIHW (2021).

[16] Nakanishi K Goto W Ishihara A Tauchi J Kashiwagi S Amano R [A case of synchronous double cancer including borderline resectable pancreatic body cancer and breast carcinoma with osseous/cartilaginous differentiation treated with neoadjuvant chemotherapy and radical resection]. Gan To Kagaku Ryoho. (2021) 48(13):2005–7. 10.1159/000477758 35045475

[17] Takada K Kashiwagi S Amano R Goto W Asano Y Ohira G [A case of male breast cancer suspected of breast metastasis from pancreatic cancer]. Gan To Kagaku Ryoho. (2018) 45(13):1857–9. PMID: 30692377 30692377

[18] Kim JS Chung CY Park HC Myung DS Cho SB Lee WS Synchronous quadruple primary tumors of thyroid, breast, pancreas, and stomach: a case report. Anticancer Res. (2013) 33(5):2135–8. 23645766

[19] Morganti AG Calista F Mignogna S Macchia G Deodato F Scambia G Synchronous appearance of male breast cancer and pancreatic cancer 15 years after the diagnosis of testicular cancer–report of a case. J BUON. (2008) 13(3):421–4. 10.1097/SMJ.0b013e31816c01a9 18979560

[20] Unek IT Alacacioglu A Tarhan O Sevinc AI Oztop I Sagol O Synchronous male carcinoma of the breast, exocrine pancreas, and prostate. South Med J. (2008) 101(5):567–8. 10.1097/SMJ.0b013e31816c01a9 18458635

[21] Khorana AA Mangu PB Berlin J Engebretson A Hong TS Maitra A Potentially curable pancreatic cancer: american society of clinical oncology clinical practice guideline. J Clin Oncol. (2016) 34(21):2541–56. 10.1200/JCO.2016.67.5553 27247221

[22] Tempero MA Malafa MP Al-Hawary M Behrman SW Benson AB Cardin DB Pancreatic adenocarcinoma, version 2.2021, NCCN clinical practice guidelines in oncology. J Natl Compr Canc Netw. (2021) 19(4):439–57. 10.6004/jnccn.2021.0017 33845462

[23] Ducreux M Cuhna AS Caramella C Hollebecque A Burtin P Goere D Cancer of the pancreas: ESMO Clinical Practice Guidelines for diagnosis, treatment and follow-up. Ann Oncol. (2015) 26(Suppl 5):v56–68. 10.1093/annonc/mdv295 26314780

[24] Cardoso F Kyriakides S Ohno S Penault-Llorca F Poortmans P Rubio IT Early breast cancer: ESMO clinical practice guidelines for diagnosis, treatment and follow-updagger. Ann Oncol. (2019) 30(8):1194–220. 10.1093/annonc/mdz173 31161190

[25] Korde LA Somerfield MR Carey LA Crews JR Denduluri N Hwang ES Neoadjuvant chemotherapy, endocrine therapy, and targeted therapy for breast cancer: ASCO guideline. J Clin Oncol. (2021) 39(13):1485–505. 10.1200/JCO.20.03399 33507815 PMC8274745

[26] Gradishar WJ Moran MS Abraham J Aft R Agnese D Allison KH NCCN guidelines(R) insights: breast cancer, version 4.2021. J Natl Compr Canc Netw. (2021) 19(5):484–93. 10.6004/jnccn.2021.0023 34794122

[27] Burstein HJ Curigliano G Thurlimann B Weber WP Poortmans P Regan MM Customizing local and systemic therapies for women with early breast cancer: the St. Gallen International Consensus Guidelines for treatment of early breast cancer 2021. Ann Oncol. (2021) 32(10):1216–35. 10.1016/j.annonc.2021.06.023 34242744 PMC9906308

[28] Rastogi P Anderson SJ Bear HD Geyer CE Kahlenberg MS Robidoux A Preoperative chemotherapy: updates of National Surgical Adjuvant Breast and Bowel Project Protocols B-18 and B-27. J Clin Oncol. (2008) 26(5):778–85. 10.1200/JCO.2007.15.0235 18258986

[29] Janssen QP Buettner S Suker M Beumer BR Addeo P Bachellier P Neoadjuvant FOLFIRINOX in patients with borderline resectable pancreatic cancer: a systematic review and patient-level meta-analysis. J Natl Cancer Inst. (2019) 111(8):782–94. 10.1093/jnci/djz073 31086963 PMC6695305

[30] Conroy T Hammel P Hebbar M Ben Abdelghani M Wei AC Raoul J-L FOLFIRINOX or gemcitabine as adjuvant therapy for pancreatic cancer. N Engl J Med. (2018) 379(25):2395–406. 10.1056/NEJMoa1809775 30575490

[31] Peto R Davies C Godwin J Gray R Pan HC Clarke M Comparisons between different polychemotherapy regimens for early breast cancer: meta-analyses of long-term outcome among 100,000 women in 123 randomised trials. Lancet. (2012) 379(9814):432–44. 10.1016/S0140-6736(11)61625-5 22152853 PMC3273723

[32] Broglio KR Quintana M Foster M Olinger M McGlothlin A Berry SM Association of pathologic complete response to neoadjuvant therapy in HER2-positive breast cancer with long-term outcomes: a meta-analysis. JAMA Oncol. (2016) 2(6):751–60. 10.1001/jamaoncol.2015.6113 26914222

[33] Moja L Tagliabue L Balduzzi S Parmelli E Pistotti V Guarneri V Trastuzumab containing regimens for early breast cancer. Cochrane Database Syst Rev. (2012) 4:CD006243. 10.1002/14651858.CD006243.pub2 PMC6718210 22513938

[34] Spring LM Gupta A Reynolds KL Gadd MA Ellisen LW Isakoff SJ Neoadjuvant endocrine therapy for estrogen receptor-positive breast cancer: a systematic review and meta-analysis. JAMA Oncol. (2016) 2(11):1477–86. 10.1001/jamaoncol.2016.1897 27367583 PMC5738656

[35] Palmieri C Cleator S Kilburn LS Kim SB Ahn SH Beresford M NEOCENT: a randomised feasibility and translational study comparing neoadjuvant endocrine therapy with chemotherapy in ER-rich postmenopausal primary breast cancer. Breast Cancer Res Treat. (2014) 148(3):581–90. 10.1007/s10549-014-3183-4 25395314

[36] Alba E Calvo L Albanell J De la Haba JR Arcusa Lanza A Chacon JI Chemotherapy (CT) and hormonotherapy (HT) as neoadjuvant treatment in luminal breast cancer patients: results from the GEICAM/2006-03, a multicenter, randomized, phase-II study. Ann Oncol. (2012) 23(12):3069–74. 10.1093/annonc/mds132 22674146

[37] Shimmura H Kuramochi H Jibiki N Katagiri S Nishino T Araida T. Dramatic response of FOLFIRINOX regimen in a collision pancreatic adenocarcinoma patient with a germline BRCA2 mutation: a case report. Jpn J Clin Oncol. (2019) 49(11):1049–54. 10.1093/jjco/hyz141 31612916

[38] Hurvitz SA Martin M Symmans WF Jung KH Huang CS Thompson AM Neoadjuvant trastuzumab, pertuzumab, and chemotherapy versus trastuzumab emtansine plus pertuzumab in patients with HER2-positive breast cancer (KRISTINE): a randomised, open-label, multicentre, phase 3 trial. Lancet Oncol. (2018) 19(1):115–26. 10.1016/S1470-2045(17)30716-7 29175149

[39] Mayer EL Dueck AC Martin M Rubovszky G Burstein HJ Bellet-Ezquerra M Palbociclib with adjuvant endocrine therapy in early breast cancer (PALLAS): interim analysis of a multicentre, open-label, randomised, phase 3 study. Lancet Oncol. (2021) 22(2):212–22. 10.1016/S1470-2045(20)30642-2 33460574

[40] Loibl S Marme F Martin M Untch M Bonnefoi H Kim SB Palbociclib for residual high-risk invasive HR-positive and HER2-negative early breast cancer-the penelope-B trial. J Clin Oncol. (2021) 39(14):1518–30. 10.1200/JCO.20.03639 33793299

[41] Khan QJ O’Dea A Bardia A Kalinsky K Wisinski KB O’Regan R Letrozole + ribociclib versus letrozole + placebo as neoadjuvant therapy for ER+ breast cancer (FELINE trial). J Clin Oncol. (2020) 38(15_suppl):505–5. 10.1200/JCO.2020.38.15_suppl.505

[42] Gil-Gil M Alba E Gavilá J de la Haba-Rodríguez J Ciruelos E Tolosa P The role of CDK4/6 inhibitors in early breast cancer. Breast. (2021) 58:160–9. 10.1016/j.breast.2021.05.008 34087775 PMC8184648

[43] Hurvitz SA Martin M Press MF Chan D Fernandez-Abad M Petru E Potent cell-cycle inhibition and upregulation of immune response with abemaciclib and anastrozole in neoMONARCH, phase II neoadjuvant study in HR(+)/HER2(-) breast cancer. Clin Cancer Res. (2020) 26(3):566–80. 10.1158/1078-0432.CCR-19-1425 31615937 PMC7498177

[44] Johnston SRD Harbeck N Hegg R Toi M Martin M Shao ZM Abemaciclib combined with endocrine therapy for the adjuvant treatment of HR+, HER2-, node-positive, high-risk, early breast cancer (monarchE). J Clin Oncol. (2020) 38(34):3987–98. 10.1200/JCO.20.02514 32954927 PMC7768339

[45] Perri G Prakash L Qiao W Varadhachary GR Wolff R Fogelman D Response and survival associated with first-line FOLFIRINOX vs gemcitabine and nab-paclitaxel chemotherapy for localized pancreatic ductal adenocarcinoma. JAMA Surg. (2020) 155(9):832–9. 10.1001/jamasurg.2020.2286 32667641 PMC7364337

[46] NSW, C.I. Breast metastatic cARBOplatin and gemcitabine. (2021). Available from: https://www.eviq.org.au/medical-oncology/breast/metastatic/1324-breast-metastatic-carboplatin-and-gemcitabine#indications-and-patient-population

[47] de Gregorio A Häberle L Fasching PA Müller V Schrader I Lorenz R Gemcitabine as adjuvant chemotherapy in patients with high-risk early breast cancer—results from the randomized phase III SUCCESS-A trial. Breast Cancer Res. (2020) 22(1):111. 10.1186/s13058-020-01348-w 33097092 PMC7583247

