# Supplementary material for: Synchronous Operable Pancreatic and Breast Cancer Without Genetic Mutation: A Literature Review and Discussion
Source: Front Surg. 2022 Jun 24;9:858349. doi: 10.3389/fsurg.2022.858349 (PMC9263594; doi:10.3389/fsurg.2022.858349)
Supplement: Supplementary file 1 [file Table_1_v1.docx]

**Table 1. Summary of 6 papers discussing synchronous breast and pancreatic cancers**

| **Authors, Year** | **Case** | **Breast Cancer** | **Pancreatic cancer** | **Treatment** | **Outcome** |
| --- | --- | --- | --- | --- | --- |
| Nakanishi et al[16], 2021 | 78y female - synchronous left breast cancer and pancreatic cancer | 48mm IDC, with nodal involvement, NFI  (T2N1M0) | 30 mm borderline resectable pancreatic body adenocarcinoma | 1. NAC: gemcitabine and nab-paclitaxel. Stable breast cancer and partial response to pancreatic cancer 2. Pancreatosplenectomy with portal vein and gastroduodenal artery resection and reconstruction + left mastectomy with axillary lymph node dissection 3. Flurouracil, epirubicin and cyclophosphamide | No recurrence noted |
| Takada et al[17],  2018 | 67y male - synchronous left breast and pancreatic cancer (with liver and peritoneal metastases) | 28mm, IDC,  TNBC  (T2N0M0) | 40mm unresectable pancreatic tail cancer | 1. Palliation due to hepatic failure secondary to pancreatic tumour growth | Died |
| Castro et al[5],  2016 | 41y female - synchronous locally advanced left breast cancer and pancreatic cancer, BRCA2 mutation | 75mm Grade 2 IDC (ER/PR+, HER2-) with  HG-DCIS, 8/23 LN  (T3N2aM0) | 26mm resectable pancreatic tail adenocarcinoma | 1. Left modified radical mastectomy 2. Laparoscopic, hand assisted splenectomy, distal pancreatectomy, lymphadenectomy and bilateral salpingo-oophorectomy 3. Adjuvant chemotherapy: doxorubicin-cisplatin followed by gemcitabine-NAB paclitaxel 4. Radiotherapy (post-mastectomy and upper abdominal) + aromatase inhibitor 5. For consideration of Olaparib therapy | Alive at time of publication |
| Kim et al[18],  2013 | 73y female - synchronous left breast cancer, pancreatic cancer, papillary carcinoma of the thyroid and GIST. | Multiple IDC, NFI  (TxNxMx) | 18mm unresectable pancreatic body adenocarcinoma with ductal dilatation and coealic axis invasion | 1. Patient refused aggressive treatment including surgery 2. Agreed for treatment of the breast cancer and GIST (hormone therapy and chemotherapy): Letrozole + imatinib 3. Supportive treatment for thyroid and pancreatic cancer | Died 8 months after diagnosis |
| Unek et al[20],  2008 | 50y male - synchronous right breast cancer and pancreatic cancer.  Background of previously treated mixed germ cell testicular tumour 15 years prior | 11mm Grade 1 IDC, ER+, PR-, HER2-, LVI+  (T4bNxM0) | 25mm resectable pancreatic head adenocarcinoma | 1. Surgery: Whipple procedure and right mastectomy 2. Radiotherapy + Chemotherapy  - Radiotherapy: to pancreatic region and chest wall - Chemotherapy: 5-Fu + Gemcitabine. Patient refused after 6 cycles due to metastatic spread | Died 10 months after diagnosis |
| Morganti et al[19], 2008 | 69y male - 2 primary right breast cancers and primary pancreatic cancer with liver metastasis  Two months post starting therapy for prostatic adenocarcinoma | 25mm + 15mm Grade 2 IDC, 0/25 LN  (T2N0M0) | 30mm unresectable pancreatic body and tail adenocarcinoma with vascular invasion with liver metastases | 1. Total right mastectomy 2. Chemotherapy: carboplatin-taxotere, 15 cycles 3. Zolidronic acid, monthly, 8 cycles 4. LHRH for prostate cancer continued 5. Chemotherapy second regimen due to disease progression: gemcitabine and oxaliplatin 6. Worsening ascitic effusion with pain, dyspnoea and haematologic toxicity. Regimen changed to gemcitabine alone | Died 17 months after diagnosis |

*5-Fu Fluorouracil, ER estrogen receptor, GIST Gastrointestinal Stromal Tumour, HG-DCIS high-grade ductal carcinoma in-situ, IDC invasive ductal carcinoma, LN lymph nodes, LVI lymphovascular invasion NAC neoadjuvant chemotherapy, NFI no further information, PR progesterone receptor, TNBC triple negative breast cancer.*
